# Contrasting resistance patterns to type I and II pyrethroids in two major arbovirus vectors *Aedes aegypti* and *Aedes albopictus* in the Republic of the Congo, Central Africa

**DOI:** 10.1186/s40249-020-0637-2

**Published:** 2020-03-02

**Authors:** Basile Kamgang, Theodel A. Wilson-Bahun, Aurelie P. Yougang, Arsene Lenga, Charles S. Wondji

**Affiliations:** 1 Centre for Research in Infectious Diseases, Department of Medical Entomology, PO Box 15391, Yaoundé, Cameroon; 2 Faculty of Science and Technology, Marien Ngouabi University, Brazzaville, Republic of the Congo; 3 Department of Animal Biology, Faculty of Sciences, University of Yaoundé I, Yaoundé, Cameroon; 4 Vector Biology Department, Liverpool School of Tropical Medicine, Liverpool, UK

**Keywords:** *Aedes aegypti*, *Aedes albopictus*, Insecticide resistance, Resistance mechanism, Republic of the Congo

## Abstract

**Background:**

In the Republic of Congo, with two massive outbreaks of chikungunya observed this decade, little is known about the insecticide resistance profile of the two major arbovirus vectors *Aedes aegypti* and *Aedes albopictus*. Here, we established the resistance profile of both species to insecticides and explored the resistance mechanisms to help Congo to better prepare for future outbreaks.

**Methods:**

Immature stages of *Ae. aegypti* and *Ae. albopictus* were sampled in May 2017 in eight cities of the Republic of the Congo and reared to adult stage. Larval and adult bioassays, and synergist (piperonyl butoxide [PBO]) assays were carried out according to WHO guidelines. F1534C mutation was genotyped in field collected adults in both species and the polymorphism of the sodium channel gene assessed in *Ae. aegypti*.

**Results:**

All tested populations were susceptible to temephos after larval bioassays. A high resistance level was observed to 4% DDT in both species countrywide (21.9–88.3% mortality). All but one population (*Ae. aegypti* from Ngo) exhibited resistance to type I pyrethroid, permethrin, but showed a full susceptibility to type II pyrethroid (deltamethrin) in almost all locations. Resistance was also reported to 1% propoxur in *Ae. aegypti* likewise in two *Ae. albopictus* populations (Owando and Ouesso), and the remaining were fully susceptible. All populations of both species were fully susceptible to 1% fenitrothion. A full recovery of susceptibility was observed in *Ae. aegypti* and *Ae. albopictus* when pre-exposed to PBO and then to propoxur and permethrin respectively. The F1534C *kdr* mutation was not detected in either species. The high genetic variability of the portion of sodium channel spanning the F1534C in *Ae. aegypti* further supported that knockdown resistance probably play no role in the permethrin resistance.

**Conclusions:**

Our study showed that both *Aedes* species were susceptible to organophosphates (temephos and fenitrothion), while for other insecticide classes tested the profile of resistance vary according to the population origin. These findings could help to implement better and efficient strategies to control these species in the Congo in the advent of future arbovirus outbreaks.

## Background

Dengue virus (DENV), Zika virus (ZIKV), yellow fever virus (YFV) and chikungunya virus (CHIKV) are *Aedes*-borne viruses of medical concern in tropical and subtropical regions. During the last two decades, diseases caused by these viruses are increasingly reported in several regions of the world including in Central Africa [1–10] where the epidemics were formerly considered as scarce (apart for YFV). These diseases are transmitted to humans through the bite of an infected mosquito belonging to the *Aedes* genus. Both urban vectors, *Aedes aegypti* Linnaeus 1762 and *Aedes albopictus* (Skuse) 1894 are well established in Africa where *Ae. aegypti* is native [11]. *Ae albopictus,* which originated from South-East Asia forests, was reported for the first time in Central Africa in the early 2000s in Cameroon [12] and has since progressively colonized almost all countries in the region including the Republic of the Congo where it tends to supplant the resident species *Ae. aegypti* in sympatric areas [13–16]. During the chikungunya outbreak reported in the Republic of the Congo in 2011 with more than 11 000 cases, CHIKV was detected in *Ae. aegypti* and *Ae. albopictus* [6, 17]. *Aedes albopictus* was suspected as the main vector during the recent chikungunya outbreak reported in 2019 in the Republic of the Congo [7]. It was demonstrated that both *Ae. aegypti* and *Ae. albopictus* from Brazzaville (Congo) are able to transmit YFV [18].

In the absence of effective vaccines (apart for YFV) and specific treatments against these viruses, vector control remains the cornerstone to prevent and control outbreaks. Existing vector control strategies include destruction of breeding sites and insecticide-based interventions. Indeed, the use of larvicides to treat water storage containers such as barrels and space spraying of adulticides in emergency situations can help to reduce the density of *Aedes* mosquitoes [19, 20]. Unfortunately, the emergence of insecticide resistance significantly hampers the efficacy of insecticides to control pests. Thus, many vector control programmes are facing the challenge from the development of insecticide resistance in *Ae. aegypti* and *Ae. albopictus.* Both major vectors have been found to be resistant to several classes of insecticides in different regions across the world with significant variation according to the population’s origin and the insecticide class including pyrethroids, organophosphates and organochlorines [21–27].

Insecticide resistance in mosquitoes is primarily associated with two major mechanisms: enhanced expression of detoxification enzymes (metabolic resistance) and insensitivity of target sites (target-site resistance) [28, 29]. Target site resistance is caused by mutations in target genes such as the acetylcholinesterase (*Ace-1*), the *GABA* receptor and the voltage-gated sodium channel (*VGSC*) causing knockdown resistance (*kdr*). One of the most important target site resistance for mosquitoes is *VGSC* as it confers resistance to both pyrethroids and dichlorodiphenyltrichloroethane (DDT). To date, 11 *kdr* mutations in *VGSC* domain I–IV have been identified in *Ae. aegypti* around the world and the association between F1534C, V1016G, I1011M and V410 L mutations and pyrethroid resistance has been established [27, 30, 31]. In Africa only 1534 and 1016 mutations have been previously reported in Burkina-Faso [31] and Ghana [24] in *Ae. aegypti*. For *Ae. albopictus*, four *VGSC* mutations have been found with only the F1534S variant been shown to be moderately associated with resistance to DDT and pyrethroids [27]. On the other hand, metabolic resistance through overexpression of detoxification genes is a common resistance mechanism in both *Ae. aegypti* and *Ae. albopictus*. The three primary enzyme families responsible for insecticide resistance in mosquitoes are the monooxygenases (cytochrome P450s), glutathione S-transferases (*GSTs*) and carboxylesterases (*COEs*) [29, 32]. In Central Africa, data on insecticide resistance in *Ae. aegypti* and *Ae. albopictus* are very scarce apart from a preliminary studies performed in Cameroon [21, 23] and Central African Republic [22]. Unfortunately, no data in this regard is available in the Republic of the Congo although the country has experienced two major chikungunya outbreaks in this decade. To fill this important knowledge gap, we established the insecticide resistance profile of *Ae. aegypti* and *Ae. albopictus* from different locations in the country and explored the potential resistance mechanisms involved to prepare the Republic of the Congo to better respond in the advent of future outbreaks.

## Methods

### Mosquito collection

**Fig. 1:**
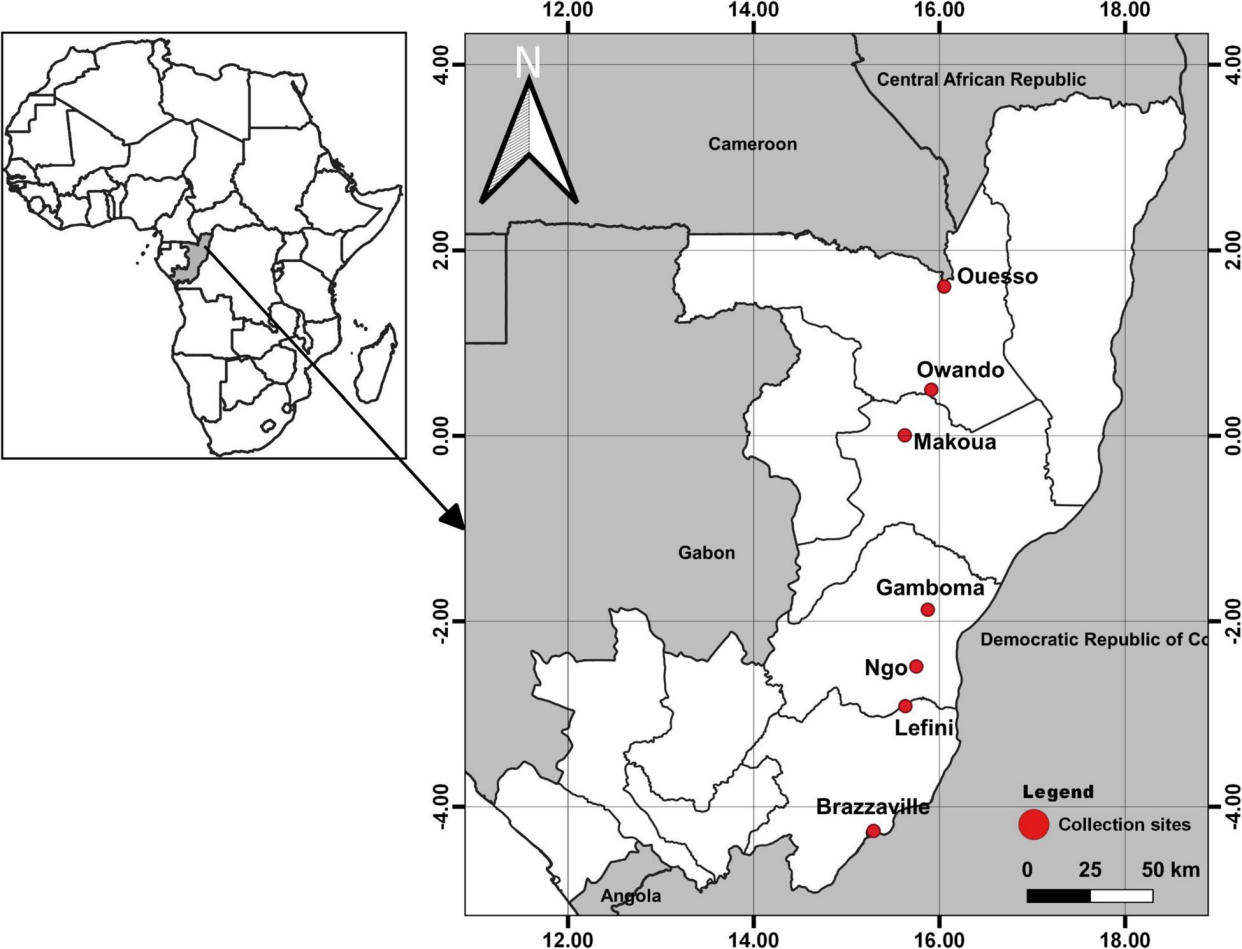
Map of the Republic of the Congo showing the sampling sites

Immature stages of *Ae. aegypti* and *Ae. albopictus* were sampled in May 2017 corresponding to the long rainy season in eight localities of the Republic of the Congo (Fig. 1): Brazzaville (S 4°15′36″ E 15°17′23″), Lefini (S 2°54′58“ E 15°37’56”), Ngo (S 2°29′14“ E 15°45’00”), Gamboma (S 1°52′27“ E 15°52’25”), Owando (S 0°29′42“ E 15°54’41”), Makoua (S 0°00′23“ E 19°37’33”) and Ouesso (N 1°36′35“ E 16°02’58”). Detailed characteristics of each collection site are presented in previous studies [13]. In each location, mosquitoes were collected in peri-urban and downtown at a minimum of 20 containers per site. Larvae/pupae of *Aedes* mosquitoes were transported to an insectary and pooled together according to the city and maintained until they emerged as adults before morphological identification using a suitable taxonomic key [33]. Adult mosquitoes were pooled according to location and species, and reared in controlled conditions at 28 ± 1 °C under 12 h dark:12 h light cycle and 80 ± 10% relative humidity until G1/G2 generation. Three reference susceptible strains were used as controls: the *Ae. aegypti* New Orleans strain, *Ae. aegypti* Benin strain and the *Ae. albopictus* susceptible strain from the Malaysia Vector Control Research Unit (VCRU).

### Insecticides susceptibility tests

#### Larval bioassays

Larval bioassays were performed according to standard WHO guidelines [34, 35] using F1/F2 larvae. The susceptibility of larvae was evaluated against technical-grade temephos (97.3%; Sigma Aldrich-Pestanal®, Germany). First, stock solutions and serial dilutions were prepared in 95% ethanol and stored at 4 °C. Five doses of concentration ranging from 0.0025 to 0.006 mg/L have been used for the assay. Eighty to 100 larvae per concentration (with three to four replicates, depending on the sample and the number of larvae available) were tested. Third late instar larvae of each species were placed in plastic cups containing 99 ml of tap water, and 1 ml of insecticide solution at the required concentration was added. Control groups were run systematically with larvae exposed to 1 ml of ethanol. No food was provided to larvae during the bioassays, which were run at 28 ± 2 °C and 80 ± 10% relative humidity. Mortality was scored after 24 h of exposure to the insecticide. Mortality rates were corrected with Abbott’s formula [36] when the mortality of controls was > 5%.

All data were analysed with Win DL v. 2.0 software [37]. Lethal concentrations (LC_50_ and LC_95_) were estimated with their 95% confidence intervals (*CI*s). Resistance ratios (RR_50_ and RR_95_) were calculated by comparing the LC_50_ and LC_95_ for each species with those of susceptible strain, as RR_50_ = LC_50_ of studied population/LC_50_ susceptible strain and RR_95_ = LC_95_ of studied population/LC_95_ reference strain. A mosquito population was considered susceptible when RR_50_ was less than 2, potentially resistant when RR_50_ was between 2 and 5, and resistant when RR_50_ was over 5 [23].

#### Adult bioassays

Insecticide susceptibility bioassays were performed with 3 to 5 day old unfed *Ae. aegypti* and *Ae. albopictus* mosquitoes according to the standard WHO guideline [35]. Six *Ae. albopictus* populations and two *Ae. aegypti* populations were used for the assays. Four replicates of 20 to 25 mosquitoes from G1/G2 generation per tubes were tested to five insecticides: 4% DDT (organochlorine), 1% propoxur (carbamate), 1% fenitrothion (organophosphate), 0.05% deltamethrin and 0.025% permethrin (pyrethroids). Insecticide-impregnated papers were supplied by Liverpool School of Tropical Medicine. Mortality was recorded 24 h later and mosquitoes alive and dead after exposure 24 h were stored in RNAlater (Sigma, Netherland) and silica gel respectively.

#### Synergist assay

In order to investigate the potential role of oxidases in the metabolic resistance mechanism, synergist assay was performed when the number of mosquitoes permitted using 4% piperonyl butoxide (PBO). Three–five day-old adults were pre-exposed for 1 h to PBO-impregnated papers and after that immediately exposed to the selected insecticide. Mortality was scored 24 h later and compared to the results obtained with each insecticide without synergist according to the WHO standards [35].

### Investigating of F1534C mutation using allele specific PCR

Genomic DNA was extracted from around 30 individuals (G0) of *Ae. aegypti* and *Ae. albopictus* per populations using the Livak protocol ([38]. These DNA were used to genotype the F1534C mutation which was found mostly widespread across the world in *Ae. aegypti* and associated with type I pyrethroid resistance and DDT resistance. Experiments were performed using allele specific (AS) PCR assays previously described [39]. As the genomic sequence of the sodium channel gene spanning the IIIS6 segment between both species is highly conserved, we employed the same AS-PCR previously designed for the F1534C variation in *Ae. aegypti* [39] also for *Ae. albopictus*. Each PCR was performed using a Gene Touch thermal cycler (Bulldog Bio, Portsmouth, USA) in a 15 μl volume containing: 1 μl of DNA sample, 0.4 units of Kapa *Taq* DNA polymerase, 0.12 μl of 25 mmol/L dNTPs (0.2 mmol/L), 0.75 μl of 25 mmol/L MgCl_2_ (1.5 mmol/L), 1.5 μl of 10 × PCR buffer (1×), 0.51 μl of each primers (0.34 mmol/L). The amplification consisted of 95 °C for a 5 min heat activation step, followed by 35 cycles of 94 °C for 30 s, 55 °C (60 °C for *Ae. albopictus*) for 30 s and 72 °C for 45 s with a 10 min final extension step at 72 °C. PCR products were detected by agarose gel electrophoresis in Tris-Acid-EDTA buffer (TAE). The 3% gel was prepared with Midori green, staining dye, and visualized with the aid of UV light.

### Polymorphism of the voltage-gated sodium channel (*VGSC*) gene in *Ae. aegypti*

To assess the polymorphism of the *VGSC* gene and detect possible signatures of selection, a fragment of this gene spanning the F1534C mutation (a part of segment 6 of Domain III) was amplified and sequenced in 30 G_0_ field collected mosquitoes from three locations in the Republic of the Congo. PCR reactions were carried out using 10 pmol of each primer (aegSCF7: GAGAACTCGCCGATGAACTT and aegSCR7: GACGACGAAATCGAACAGGT) and 20 ng of genomic DNA as template in 15 μl reactions containing 1 × Kapa *Taq* buffer, 0.2 mmol/L dNTPs, 1.5 mmol/L MgCl_2_, 1 U Kapa *Taq* (Kapa biosystems). The cycle conditions were 95 °C for 5 min and 35 cycles of 94 °C for 30 s, 57 °C for 30 s and 72 °C for 1 min, followed by a final extension step of 72 °C for 10 min. The samples were purified using Exo-SAP (New England Biolab, UK) protocol according to manufacturer recommendations and sent for sequencing in Centre for Genomic Research at the University of Liverpool. The sequences were visualized and corrected when necessary using BioEdit software version 7.0.5.3 and aligned using ClustalW [40]. DnaSP v5.10 [41] was used to define the haplotype phase and the genetic parameters including number of haplotypes (h), the number of polymorphism sites (S), haplotype diversity (Hd) and nucleotide diversity (π). The statistical tests of Tajima [42], Fu and Li [43] were estimated with DnaSP in order to establish non-neutral evolution and deviation from mutation-drift equilibrium. A haplotype network was built using the TCS program [44] to further assess the potential connection between haplotypes. A maximum likelihood tree of the sequences obtained and reference sequences from Brazil and Thailand was constructed using MEGA 7.0 [45].

## Results

### Larval bioassay

Due to the limited number of larvae available, larval bioassays were performed for two *Ae. aegypti* populations from Ngo and Brazzaville, and one *Ae. albopictus* population from Brazzaville. Analysis revealed that for both *Aedes* species and populations tested, RR_50_ and RR_95_ were less than 2 suggesting that *Ae. aegypti* and *Ae. albopictus* of these locations are susceptible to temephos (Table 1)*.*

**Table 1:** Larval bioassays of *Aedes aegypti* and *Ae. albopictus* with temephos

Strains and sites	*N*	LC_50_ (mg/L) (95% *CI*)	RR_50_	LC_95_ (mg/L) (95% *CI*)	RR_95_
*Ae. aegypti*					
Reference strain	531	0.00268 (0.0025–0.0028)		0.00463 (0.0043–0.0051)	
Ngo	410	0.00259 (0.0024–0.0028)	0.96	0.00443 (0.0040–0.0051)	0.95
Brazzaville	423	0.00413 (0.004–0.0043)	1.54	0.00528 (0.0050–0.0057)	1.14
*Ae. albopictus*					
Reference strain	574	0.00310 (0.0013–0.0041)		0.0068 (0.0051–0.0209)	
Brazzaville	506	0.00412 (0.0027–0.0062)	1.33	0.0069 (0.0043–0.0113)	1.02

### Insecticide resistance profile in adults *Aedes*

Assays performed with laboratory susceptible strains confirmed that *Ae. albopictus* (VCRU), *Ae. aegypti* (New Orleans) and *Ae. aegypti* (Benin) were totally susceptible to insecticides tested except to DDT for which 80.68% (VCRU strain) and 98.75% (New Orleans strain) mortality rates were found, respectively. The mortality rate in controls was less than 5%.

### Resistance pattern for *Ae. aegypti*

**Fig. 2:**
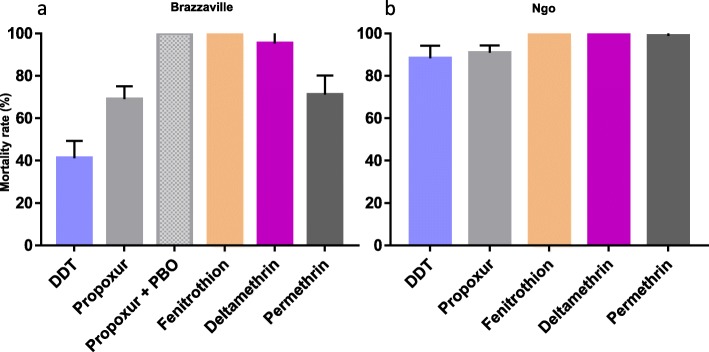
Mortality rates of adult *Aedes aegypti* from the Republic of the Congo 24 h after exposure to insecticides alone or with 1 h pre-exposure to synergist. **a**, Brazzaville; **b**, Ngo. Error bars represent standard error of the mean. DDT, Dichlorodiphenyltrichloroethane. PBO, Piperonyl butoxide

Two *Ae. aegypti* populations from Brazzaville (the major city of the country) and Ngo were tested for resistance to five insecticides (Fig. 2). In Brazzaville population, resistance was observed against the organochlorine DDT with a low mortality rate of 41.2%. Resistance was also observed against pyrethroids, notably permethrin (type I) with 71.0% mortality registered whereas mortality was higher (95.3%) for deltamethrin (Type II). Noticeably, this *Ae. aegypti* population of Brazzaville is resistant to carbamates with 69.0% mortality for propoxur. However, full susceptibility to the organophosphate fenitrothion was reported in this population in line with the susceptibility observed at the larval stage against the other organophosphate, temephos. The other population from Ngo displayed a moderate resistance level to DDT (88.3%) and a probable resistance to propoxur (91.0%). In contrast to Brazzaville, the Ngo population was fully susceptible to both types of pyrethroids highlighting a variation of susceptibility profile of *Ae. aegypti* across Congo. It also exhibited a full susceptibility toward the organophosphate fenitrothion (Additional file 1).

### Resistance pattern for *Ae. albopictus*

**Fig. 3:**
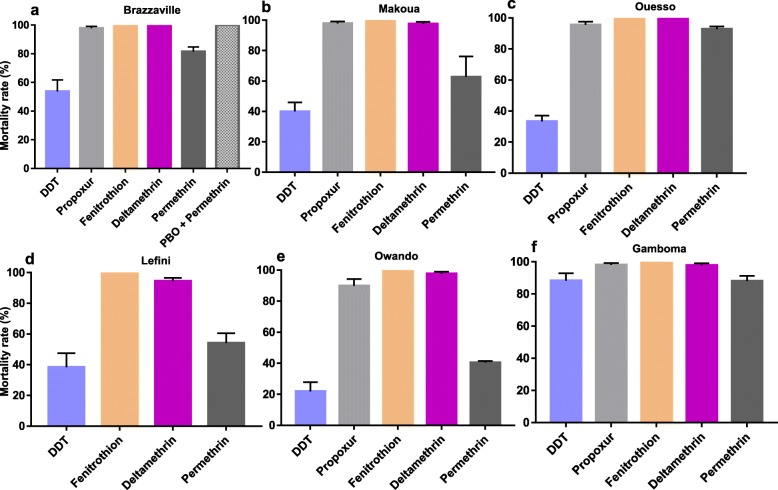
Mortality rates of adult *Aedes albopictus* from the Republic of the Congo 24 h after exposure to insecticides alone or with 1 h pre-exposure to synergist. **a**, Brazzaville; **b**, Makoua; **c**, Ouesso; **d**, Leffini; **e**, Owando; **f**, Gamboma. Error bars represent standard error of the mean. DDT, Dichlorodiphenyltrichloroethane. PBO, Piperonyl butoxide

For *Ae*. *albopictus*, six populations were analysed: Brazzaville, Lefini, Ouesso, Gamboma, Makoua and Owando (Fig. 3). Analysis revealed that all the populations tested were resistant to DDT with the mortality rate ranging from 21.9% in Owando to 88.3% in Gamboma. A similar pattern was observed for type I pyrethroid permethrin with mortality rates varying from 40.5% in Owando population to 92.8% in Ouesso. However, for the type II pyrethroid deltamethrin, a probable resistance was reported in Lefini with a mortality rate of 94.4% whereas the other five populations were found susceptible with mortality rates ranging from 98.0% (Makoua, Gamboma and Owando) to 100.0% (Brazzaville and Ouesso). A moderate resistance was detected against the carbamate propoxur in Owando (90.0% mortality) and Ouesso (95.5%) whereas the remaining populations were fully susceptible. As for *Ae. aegypti* populations, a full susceptibility to the organophosphate fenitrothion was reported in all populations (Additional file 1).

### Synergist assay with PBO

Results from the synergist assay in the *Ae. albopictus* population from Brazzaville resistant to permethrin showed a full recovery of susceptibility after PBO pre-exposure (81.5 ± 3.3% mortality without PBO pre-exposure vs 100 ± 0.0% mortality after PBO pre-exposure, *P* < 0.001) suggesting that cytochrome P450 enzymes play a major role in permethrin resistance in this population.

Similar analysis for the *Ae. aegypti* population from Brazzaville showed a full recovery of susceptibility after PBO pre-exposure for the propoxur (69.0 ± 6.1% mortality without pre-exposure versus 100 ± 0.0% mortality after PBO pre-exposure, *P <* 0.001) suggesting that cytochrome P450 enzymes also play a major role in carbamate (propoxur) resistance in this population (Fig. 3).

### F1534 genotyping

After genotyping 64 specimens of *Ae. aegypti* from three locations and 136 specimens of *Ae. albopictus* from six locations no resistant individual was detected in both species with 100% homozygote F1534 mosquitoes detected.

### Genetic diversity of *VGSC* gene in *Ae. aegypti*

**Fig. 4:**
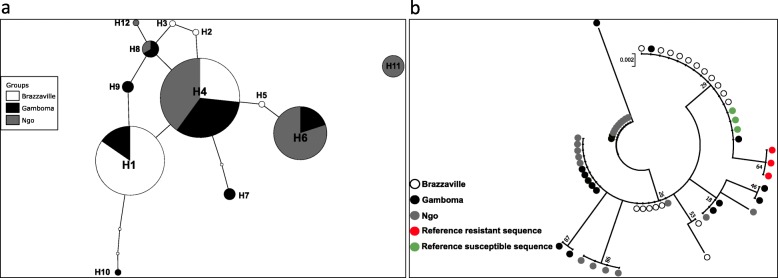
Genetic diversity of a fragment of *VGSC* gene spanning F1534C mutation of *Aedes aegypti* from the Republic of the Congo. **a**, Haplotype network showing the genealogic relationship between the twelve haplotypes detected; **b**, Maximum likelihood phylogenetic tree of the *VGSC* sequences

Twenty seven field collected *Ae. aegypti* from three locations were successfully sequenced for an 198 bp fragment of the *VGSC* gene spanning the codon 1534. Analysis confirmed the absence of F1534 mutation. Overall, a high genetic diversity is observed for this fragment with 10 polymorphic sites, 12 haplotypes, eight synonymous and two non-synonymous mutations associated with a high haplotype diversity (0.823) and relatively low nucleotide diversity (0.008) (Table 2). This high diversity is also supported by the pattern of TCS haplotype network with haplotypes separated with several mutational steps suggesting a lack of selection in this locus although three haplotypes are predominant. The predominant haplotype H4 is found in all three populations (Fig. 4a). A maximum likelihood (ML) tree of the sequences analysed confirms a high diversity with the potential three clusters (Fig. 4b). Overall, all the statistics estimated were negatives (D = − 0.79, Fs = − 3.82, and F* = − 0.19), but not statistically significant (Table 2). Negative values for these indices indicate an excess of rare polymorphisms in a population and suggest either population expansion or background selection [43].

**Table 2:** Summary statistics for the polymorphism of the *VGCS* portion in *Aedes aegypti* from the Republic of the Congo

Locality	*N*	Hp	S	HpD	syn	nonsyn	π (k)	D	Fs	F*
Brazzaville	18	5	4	0.601	3	1	0.005(0.935)	−0.588 ^ns^	−1.430 ^ns^	−0.770 ^ns^
Gamboma	16	7	8	0.875	7	1	0.011(2.133)	−0.415 ^ns^	− 1.519 ^ns^	0.121 ^ns^
Ngo	20	6	4	0.742	4	0	0.007(1.384)	0.659 ^ns^	−0.285 ^ns^	1.133 ^ns^
Total	54	12	10	0.823	8	2	0.008(1.579)	−0.792^ns^	−3.82 ^ns^	− 0.193 ^ns^

*N* Number of sequences analysed, *Hp* Number of haplotypes, *S* Number of segregating sites, *syn* Synonymous mutation, *nonsyn* Non-synonymous mutation, *HpD* Haplotypes diversity, π Nucleotide diversity, *K* Average of number of nucleotide difference; Tajima’s D and Fu’s Fs and Fu and Li F* statistics; ns, Non-significant

## Discussion

This study investigated for the first time the insecticide profile of *Ae. aegypti* and *Ae. albopictus* in the Republic of the Congo and explored the resistance mechanism involved. Analysis of larval bioassays revealed that all *Ae. aegypti* and *Ae. albopictus* samples tested were susceptible to temephos. This result is consistent with the previous result obtained in Central Africa notably in Cameroon [23], Gabon [23] and Central African Republic [22]. Nonetheless, the resistance to this compound has been reported in several countries such as in Brazil [46, 47], Malaysia [48], Thailand [49] and Carpe Verde [50] for *Ae. aegypti* and in Greece [51], Malaysia [48] and Thailand [49] for *Ae. albopictus*. Selection of the resistance results from extensive and long-term use of the product incriminated, meanwhile in our knowledge, temephos had never been used in vector control programs in Central Africa which probably explains the full susceptibility reported in both species.

Both adult populations of *Ae. aegypti* tested were resistant to DDT. A similar pattern of resistance to DDT was shown in *Ae. albopictus* populations. A decreasing susceptibility of the *Ae. aegypti* population from Brazzaville towards DDT was already mentioned in 1970s [52], suggesting that this resistance may have resulted from a continuing selection pressure on *Aedes* populations as suggested previously [22]. DDT resistance has repeatedly been reported in *Ae. aegypti* [21, 22, 48, 53] and *Ae. albopictus* [21, 54–56]. Both species exhibited a significant level of resistance against the type I pyrethroid permethrin, loss of susceptibility to this insecticide was previously reported in neighbouring countries such as Cameroon [21]. However, the fact that one population of *Ae. aegypti* remains fully susceptible suggests that pyrethroid resistance has not yet spread nation-wide in the Republic of the Congo. On the other hand, for the type II pyrethroid, deltamethrin, no resistant population was found despite a reduced susceptibility reported in some populations in both species. The striking difference of the resistance pattern in both pyrethroids tested could be due to the fact diagnostic dose used for deltamethrin 0.05% is higher than 0.03% recommended for *Aedes* [35]. The reduced susceptibility to deltamethrin and resistance to permethrin observed in both species may poses a serious threat for vector control programmes, because pyrethroids are mainly recommended for the control of adult *Aedes* mosquitoes [57, 58]. A loss of susceptibility was reported also to propoxur with moderate level of resistance in *Ae. aegypti* population from Brazzaville. Similar patterns of insecticide resistance profile found in this study were reported in several countries in Africa such as Burkina-Faso [59], Central African Republic [22], and Cameroon [21]. The source of selection driving the observed resistance to DDT and permethrin as well as the reduced susceptibility to deltamethrin and propoxur in the Republic of the Congo as in other central African countries remains unclear notably as the use of insecticides against *Ae. aegypti* and *Ae. albopictus* is limited in the region [21, 23]. As suggested previously [21, 22], domestic used of insecticides through the indoor spraying and impregnating bed nets, and agriculture use could be the main source of resistance selection in *Aedes* vectors in Central Africa. The higher resistance level to DDT observed in *Ae. aegypti* in the Republic of the Congo would be probably a consequence of the intense DDT spraying in the 1950s and 1960s as part of the malaria elimination campaign as suggested previously [21]. Meanwhile, for *Ae. albopictus* which was first reported in the Republic of the Congo in 2011 during the chikungunya outbreak in Brazzaville [6], we cannot exclude the possibility that the invading populations possessed the resistance background, as suggested previously [22, 23].

A full recovery of susceptibility to permethrin and propoxur was reported in *Ae. albopictus* and *Ae. aegypti* from Brazzaville respectively after pre-exposure to PBO synergist. This result indicates that the cytochrome P450 monooxygenases are playing the main role in the observed resistance which is consistent with previous data from the sub-region [21, 22]. None of the specimens of *Ae. aegypti* or *Ae. albopictus* genotyped possesses the 1534C allele suggesting this mutation is not currently involved in pyrethroid resistance in populations of both species in Congo. Nevertheless, this mutation known as most widely distributed in *Aedes aegypti* [27] has been detected in sample from West Africa in Ghana [24] and Burkina-Faso [59]. This mutation has also been detected in *Ae. albopictus* from several countries outside Africa like Brazil, India, Greece, Singapore and China [60]. The high genetic diversity of the *VGSC* portion spanning codon 1534 further supports the absence of *kdr* mutation in *Ae. Aegypti* in Congo as shown by the ML phylogenetic tree and TCS haplotype network. This is similar to the situation where *kdr* in absent as in *Ae. albopictus* population from Malaysia [48] or the malaria vector *Anopheles funestus* [61, 62]. It will be interesting to extend this work in other locations throughout the country, genotype other mutations such as 1016 and 410 which have been found implicated in *kdr* resistance in *Ae. aegypti* [27, 30, 31] and investigate the genes involved in metabolic resistance in both *Aedes* species.

## Conclusions

Our study showed that both *Ae. aegypti* and *Ae. albopictus* species were susceptible to organophosphates (temephos and fenitrothion), while for other insecticide classes tested the profile of resistance vary according to the population sampled. This first countrywide resistance profile to main insecticide classes in both *Aedes* vectors in the Republic of Congo should enable this country to quickly implement insecticide-based control interventions in the event of future outbreaks. The full susceptibility of both species to organophosphates at both larval and adult stages makes this insecticide class very suitable for control nation-wide.

## Supplementary information

**Additional file 1: Figure S1.** Map of the Republic of Congo showing the resistance status of *Aedes aegypti* and *Ae. albopictus* to insecticide. a, *Aedes albopictus*; b, *Ae. aegypti*.

## Data Availability

DNA sequences reported in this paper were deposited at GenBank (accession number MN823932-MN823943).

## Abbreviations

AS: Allele specific
DDT: Dichlorodiphenyltrichloroethane
DNA: Deoxyribonucleic
kdr: Knockdown resistance
LC: Lethal concentration
PBO: Piperonyl butoxide
PCR: Polymerase chain reaction
RR: Resistance ratio
VCRU: Vector control research unit
*VGSC*: Voltage-gated sodium channel
WHO: World Health Organization
