# Supplementary figures and images for: Contrasting resistance patterns to type I and II pyrethroids in two major arbovirus vectors Aedes aegypti and Aedes albopictus in the Republic of the Congo, Central Africa
Source: Infect Dis Poverty. 2020 Mar 2;9:23. doi: 10.1186/s40249-020-0637-2 (PMC7050138; doi:10.1186/s40249-020-0637-2)

## Slide 1
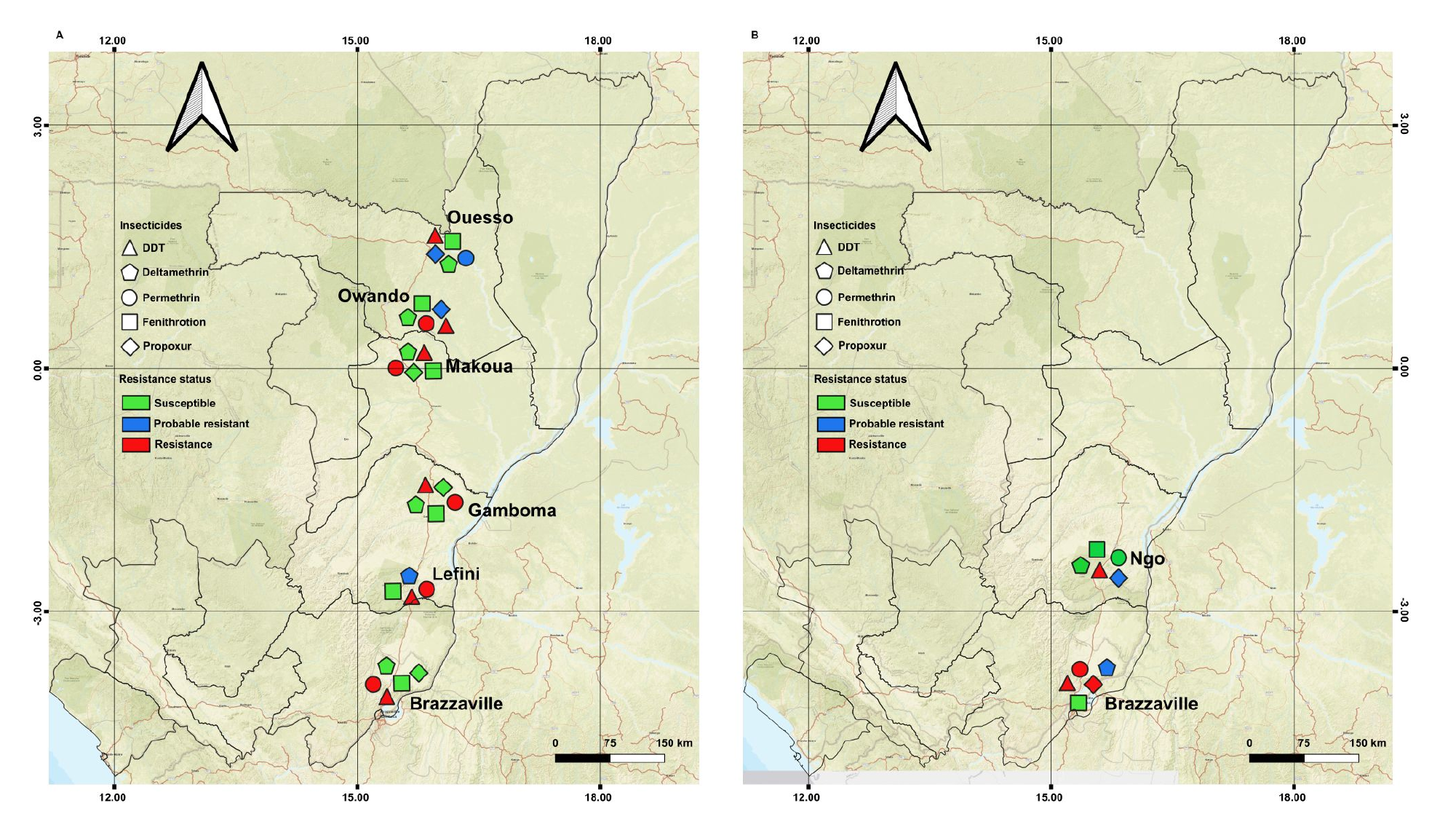

Supplement: Supplementary file 1 — Additional file 1: Figure S1. Map of the Republic of Congo showing the resistance status of Aedes aegypti and Ae. albopictus to insecticide. a, Aedes albopictus; b, Ae. aegypti. [file 40249_2020_637_MOESM1_ESM.ppt]
